# Successes and challenges of the Home-grown School Feeding Program in Sidama Region, Southern Ethiopia: a qualitative study

**DOI:** 10.1017/jns.2022.77

**Published:** 2022-09-30

**Authors:** Tsion A. Desalegn, Samson Gebremedhin, Barbara J. Stoecker

**Affiliations:** 1 School of Nutrition, Food Science and Technology, Hawassa University, Hawassa, Ethiopia; 2 School of Public Health, Addis Ababa University, Addis Ababa, Ethiopia; 3 Department of Nutritional Sciences, Oklahoma State University, Stillwater, OK, USA

**Keywords:** Barriers, Challenges, Ethiopia, Home-grown School Feeding Program (HG-SFP), Successes

## Abstract

The Home-grown School Feeding Program (HG-SFP) is a model designed to provide school meals to students using foods sourced from local markets. HG-SFP recently has been incorporated as one of the strategies of educational development in Ethiopia aiming to address hunger and food insecurity problems of school children. Yet, evaluation of the successes and challenges of the program has been limited evaluated. The purpose of the present study was to explore the successes and challenges of the SFP in Sidama Region, Southern Ethiopia. This exploratory qualitative study collected data from eight schools targeted for HG-SFP through key informant interviews and focus group discussions (FGDs). A total of sixteen FGDs and twenty-one in-depth interviews were conducted. Purposive sampling was used to include study participants based on their potential relevance in delivering in-depth information. The findings of the present study showed that HG-SFP was successful in improving class attendance and academic performance of school children. In addition, the program had a contribution in saving the parents’ money and time as a result of the food provided. With regard to SFP challenges, lack of permanent clean water provision, delay in ration delivery, poor-quality food provision, inadequate amount of food allocated for the academic year, lack of necessary infrastructure for the program, and lack of training in sanitation and hygiene for cooks were among the major challenges identified. Therefore, program challenges need high-level attention in order to make the school feeding program more successful in Sidama Region, Ethiopia.

## Introduction

School feeding programs (SFPs) are cost-effective interventions that provide nutritious foods to school children and adolescents attending school regularly in order to safeguard vulnerable children from hunger^(1,2)^. Reported benefits include avoiding hunger, improving school enrollment and attendance, improving household food security, increasing academic performance and contributing to gender equity in access to education^(3,4)^.

SFPs frequently targeted food insecure and socio-economically disadvantaged populations in almost low- and middle-income countries with the support of World Food Program (WFP)^(3)^. According to FAO, a considerable proportion of Ethiopia's population lives in a state of chronic food insecurity^(5)^. Sidama Region is among the most food-insecure areas of southern Ethiopia^(6)^. According to a recent study, the prevalence of severe food insecurity in the study area was 48 %^(7)^. Households in such areas usually find it difficult to feed the entire family because their own production of food falls short of the demand in the household. Therefore, children need to engage in some kind of activity to generate livelihood for their households. Consequently, many primary school age children in food insecure areas remain out of school.

An SFP that is connected to the local purchase of food or agricultural development is known as a Home-grown School Feeding Program (HG-SFP)^(3,8)^. Such a program helps to boost local economies^(9)^. In many high-income countries where the prevalence of malnutrition is low, school feeding (SF) is an important element of national social protection systems^(10)^. HG-SFP provides market opportunities to small-holder farmers and stimulates the economy at the community level through targeted procurement. Furthermore, the initiative promotes nutrition education and better eating habits and encourages the diversification of production with a special emphasis on local crops^(11)^.

Ministries of Agriculture, Education and Health are important supporters of HG-SFPs. In terms of agriculture, the goal is to stimulate local agriculture production by creating a market for locally grown commodities through school meals^(12)^. However, some conditions must be satisfied in order to generate the desired effects. For example, inputs such as improved seed and fertilisers for small-scale farmers in targeted districts are very important to boost production capacity^(12)^.

The HG-SFP in southern Ethiopia is a cost-effective school meal consisting of locally produced maize, beans and cracked wheat. Each school child in the targeted area should receive a daily hot meal prepared from 150 g of dry cereals and beans in different forms. A local food called ‘Nifro’ is a boiled mixture of maize, beans, vegetable oil and iodised salt. The other main menu item under the HG-SFP is called ‘Kinche’ and is made from cracked wheat with added vegetable oil and iodised salt^(13)^. The existing SFP food menu is expected to provide at least one-third of the daily energy requirements. According to the World Health Organization (WHO) recommendations, a single meal should contribute 30–45 % of the recommended daily allowance (RDA) of energy and nutrients for half-day schools and 60–75 % for full-day schools^(14)^.

In Ethiopia, a WFP-sponsored SFP was started as a pilot project in 1994 in war-affected zones in Tigray Region^(3)^. Later, SFPs were initiated in chronically food insecure districts of Afar, Amhara, Oromia, SNNPR, Somali and Tigray regions with a particular focus on districts with lower enrollment and higher gender disparity^(15)^. Then, the program expanded to other food insecure and vulnerable areas in Ethiopia, mainly in pastoralist areas and chronically food deficit highland districts to attract children to school^(15)^. The national coverage of the program has gradually increased and as of 2019 more than a million children in drought-affected areas were benefiting from SFPs^(16)^. Presently, the national nutrition program (NNP) of Ethiopia has already identified the promotion of HG-SF as a key nutrition-sensitive intervention to combat malnutrition^(17)^.

So far, only a few studies have evaluated HG-SFPs. And little is known about the successes and challenges of HG-SFPs in Ethiopia. In studies conducted in Afar and Somali regions, different challenges were identified. Among these, lack of funds for the schools and independent structure, lack of continuous resource mobilisation, lack of effective monitoring and evaluation and improper utilisation of the allocated food were among the major challenges^(18)^. Another study conducted in Jigjiga Zone, Somali regional, Ethiopia, showed that delay in ration delivery, problems with kitchen utensils, lack of storage facilities and water supply were reported as the key challenges of the program^(19)^.

Even though HG-SFP has been implemented in Sidama Region since 2005, the successes and challenges of the SFP have not yet been reported. Therefore, the purpose of the present study was aimed at discovering the successes and challenges of the SFP in the selected public schools in Sidama Region, Southern Ethiopia. The findings of the study benefit to generate information on the operation of the SFPs in the study area and create a better way to improve the program and also it helps the beneficiary students to get standard service.

## Materials and methods

### Study design

An exploratory qualitative study was conducted to assess the successes and challenges of the HG-SFP. Interview and discussion guides were used to facilitate the dialogue among selected groups and individuals from students’ parents, school directors, teachers, and zonal and district education and health office delegates. Data were gathered from the study settings from May 2018 to June 2018.

### Study setting

The study was conducted in eight second-cycle primary schools (SCPS) in four SF targeted rural districts (Borecha, Dara, Bona and Loka Abaya) of Sidama Region, Southern Ethiopia. The region is located approximately 300 km south of the national capital, Addis Ababa, and has around four million inhabitants, of whom 95 % are rural dwellers^(20)^. The region covers nearly 10 000 km^2^ area and is characterised by diverse agro-ecological features. The selected schools were enrolled into the HG-SFP since 2005.

### Sampling procedure

First, the SFP delegates from the zonal education office were contacted to give an overview of the program across all the four districts where SFPs were implemented in the region, namely Borecha district, Loka Abaya district, Dara district and Bona district. From each district, two schools were randomly selected. Totally eight schools targeted for feeding programs were included in the study. From the selected schools, stakeholders with significant roles in the SFP (students, parents, teachers, school principals and school feeding district focal) were purposely recruited through in consultation with the school principals. Accordingly, a total of sixteen focus group discussions (one FGD/school for mothers and one for students) and a total of twenty-one key informant interviews (KIIs) were conducted: two KII/school (one KII for a teacher and one KII for a school director) as well as one KII for a zonal delegate and one KII for each of the four district's education office delegates. The study included 21 participants for in-depth interviews and 144 participants for FGDs. On average, nine participants were included in one FGD. Totally sample size of this study was 165 participants based on data saturation principles.

### Data collection tools and procedure

Semi-structured discussion and interview guides were developed to assess the facilitators and barriers of the HG-SFP in the study area (Supplementary Table S1). The FGDs for in-school students and mothers and the KIIs for school teachers, school directors and education office delegates were conducted in private settings where recording was possible with little disturbance. Data were audio-taped and transferred to a personal computer to which only investigators had access. Audio-taped files were transcribed verbatim using the local language (Sidamegna) and translated to English. Each FGD and KII was transcribed before the next data collection, which enabled the capture of emerging insights into the semi-structured guide to enhance the credibility and comprehensiveness of the conversations. The data generated from FGDs and KIIs were triangulated by the principal investigator.

### Data analysis

Qualitative software (ATLAS.ti, v. 7.5.4) was used to store, manage and code all transcribed data. Transcripts were analysed using both inductive and deductive coding. Two assistants independently coded the data and in the case of difference, discussions were held to reach an agreement. Similar codes were grouped into categories that linked the codes. Then, categories were merged into themes in reference to the study objectives (Supplementary Table S2). All texts under quotation represent direct words of the study participants translated to the English language.

### Ethical considerations

The study was implemented in accordance with the Declaration of Helsinki for research involving human subjects. The study protocol was approved by Hawassa University's Ethical Review Committee with Reference No: IRB/003/10 and data were collected after taking written informed consent and assent from all subjects/patients.

## Results

### Socio-demographic characteristics

An average of nine students or mothers (ranging from 6 to 12) participated in each FGD. The age of the student participants was between 10 and 14 years and mothers were 30 and 50 years old. School teachers, school directors and zonal and district education office delegates were interviewed to complement information provided by the students and mothers. Overall, a total of 165 participants were included in the study. The successes and barriers to the HG-SFP in the study area were explored as described in detail below.

### Successes of the HG-SFP

The HG-SFP model is designed to provide cost-effective in-school meals consisting of locally produced food purchased from small-holder farmers through contract agreements between the education office and the farmers’ cooperative unions. One major success of the program has been decreasing the cost for purchase and transport of food commodities. During the in-depth interviews, education office delegates explained that the local procurement is an effective means of finding appropriate types of food at low cost with fast delivery as compared to imported food aid from donors. In addition to this, the delegates stated that the HG-SFP has benefits to the local economy by creating reliable market access for the farmers.

The other notable success of SFP is that the school children enjoy the existing HG-SFP menu. In KIIs, the school directors stated that students are very happy to eat the locally purchased food. The food was prepared by considering the number of beneficiaries based on the daily attendance sheet. Each child in a targeted school should receive the daily hot meal as foods called ‘Nifro’ or ‘Kinche’ based on the schedule. Most of the students in the FGDs explained also that students were eating the locally purchased food happily. One of the students from the FGDs explained that ‘ … we are very happy to eat the current school food. I and most of my friends didn't like the taste and the smell of the previous food’.

Another important success of the HG-SFP is improving enrollments, academic performance and absenteeism. The success of the SFP was frequently mentioned by school directors and teachers as a program which increased school enrollment and class attendance and lowered student drop-out rates, especially for girls when compared with previous years when the program had not been introduced. One of the school directors explained ‘ … Before the SFP was launched in our school, we had observed that many children would not come to our school because the children would participate in different household activities to help their parents, especially female students. Currently this situation has considerably been improved and the feeding program is very helpful to have students in the school’.

Another school director also reported that the program improved student enrollment and reduced class absenteeism to its minimum level. The opinion was captured in the following quotes from this director ‘ … I think the feeding program is an important strategy to attract students to school. Compared to the previous years, the number of students has increased and the number of students who miss the class has decreased. And even students from other kebeles frequently requested us to join this school because of the meals we provide’.

Other benefits from the program are that the children are not absent from the school because of lack of food at home, and this helps parents to save money and time to prepare food.

One mother in an FGD explained that, ‘My son is learning in this Faleqa primary school, and most of the time I don't have anything to give him at home as breakfast. Before the SFP started, my son frequently had missed the class preferring to do work for money/food than attending school’.

### Challenges for the HG-SFP

The KI and FGD participants indicated that the program had a number of challenges: lack of access to safe drinking water sources, absence of firewood, late delivery of supplies and poor quality of cereal (discoloured and damaged grain), and most of storerooms were not ventilated and were poorly constructed. Most of the schools did not have good and permanent kitchen structures. Almost all schools did not have an adequate stock of plates, cups and spoons.

Other challenges mentioned in the study area were that the whole grain cereals were dry and they took a long time to cook. As a result, it had created a high work load for cooks. In addition, cooks in the study area had not received any formal training about hygiene and sanitation since they started the job, and this was among the main challenges discussed.

Also, water scarcity is one of the main challenges that affect the SFP in the study area. Water scarcity has huge impacts on both food production and food security. Lack of enough production affects the household's food security. In food insecure area, it is difficult to feed the entire family because their own production of food is smaller than needed; this affects the right to access adequate food in all times for the household.

As most of the participants explained, 75 % of the schools did not have a water line in the compound. Even in the schools with water supply facilities, still there was a problem of water because of the water supply frequently gets interrupted for days. One of the school directors explained during a KII ‘ … we do have water supply facilities in our school. But there is mostly no water in the lines. And often time there is no water supply for about one month. When we buy water, filling 20 liter water-containing jar costs us about 5 birr (0⋅16 USD)’.

In addition, school directors complained that the farmers’ cooperative union does not deliver the food to the school in time. Due to this, some students missed school in the beginning of the academic year. One of the school directors stated that ‘ … yes, we have a problem of food delivery delay. For this year the cereals were provided to us in November. We received the food through the farmer's union’.

School directors and teachers frequently complained that the delivered food was poor in quality and lacked uniformity in the size of packs and expiry date labelling. The cause of poor-quality cereals might be a post-harvest management problem among farmers as well as poor storage facilities and management at the school. This concern was shared by a school director ‘ … we had a problem of cereal quality at the time of delivery. We received the food through the farmer's union and they delivered some already spoiled cereals bags. For example, in our school, out of 68⋅5 quintals of maize, 20 quintals of maize were already spoiled at the time of delivery. We appealed the problem to the district education office but so far we haven't got any response’.

Another director also explained during an in-depth interview: ‘ … on average more than 10 kg of waste is separated from a 50 kg cereal bag … and also the bag weight is not always constant as 50 kg, not properly sealed and expiry date is not labeled’.

Participants explained that the amount of cereals allocated for the academic year is not enough. For these reasons, they are unable to feed the children the recommended amounts. In addition, the food takes a long time to be cooked, and it consumes much firewood and water. These concerns were shared by all school directors and teachers. One of the school directors explained the following during an in-depth interview ‘ … We don't have water in our school compound, even in the near distance to the school. For these reasons we required each student to bring a bottle of water from his/her home to attend the school meal of the day. We don't have budget to buy water daily to cook food for thousands of students.  …. The food needs much water and firewood to be cooked, specially the “Nifro” because it is made from dry maize and bean’.

The result of the present study shows that the nutritional benefits of school meals are inadequate as explained by the study participants. A student from an FGD complained that the amount of the food they received was inadequate and unsatisfactory. And, this concern was shared by one of the students who explained it in the following way. ‘The food that we received in school is too small. We are unhappy with the amount’.

Another school director also explained the following during a KII. ‘ … the World Food Program trained us to provide meal prepared from 100 g dry whole grain maize, 50 g beans, 10 g vegetable oil and 5 g iodized salt per each student. But we don't provide them this amount actually because the amount of the food which we received was not enough to do this daily. Unless we make some adjustment in the amount, the food is not enough for the whole academic year’.

FGD discussants of mothers who worked as cooks mentioned that the poor-quality cereals affected their cooking by taking more time to clean and by increasing workload. This concern was shared by the school director from one of the districts who voiced a similar reflection ‘ … The problem of the quality of the cereal delivered to our school has forced us to use additional numbers of cookers in our school to separate the unwanted spoiled grain from the cereal. In average they separate more than 10 kg of waste from a 50 kg bag’.

Most of the KIs mentioned that the HG-SF menu lacks important micronutrients and is inadequate to improve the nutritional status of the students. Therefore, most of the KIs commented on HG-SF menu quality. A director from one school said the following during the interview. ‘ … The HG-SF should be delivered in better quality and quantity to improve the nutritional status of the students’.

Another director during the interview also said the following ‘including some fruit and vegetables to the school food would increase the benefit that the students get from the school food’.

## Discussion

The present study indicated that the major successes of the feeding program are its benefits for improving enrollments, academic performance and absenteeism. The SFP also might have improved the quality of education by alleviating short-term hunger. This prevents the need for children to leave the school to find food and increases attention of students in the classroom which results in better academic performance. Hunger is a barrier to learning and SFPs throughout the world have successfully addressed this hindrance and subsequently attracted children to school. Studies so far carried out in most countries indicated that a HG-SFP is one of the effective interventions to address the challenges of low school enrollment and attendance as well as poor academic performance and other issues^(1)^.

The concept of the SFP in developing countries like Ethiopia was generally aimed at improving the quality of learning of the poor through improved enrollment, attendance and academic performance^(3,4)^. Studies undertaken show multiple benefits of SF on educational outcomes that include increased enrollment and attendance, a lower drop-out rate and improved school performance^(21)^. SFPs can help to get children into school and help to keep them there, through improving enrollment and reducing absenteeism. Once the children are in school, the program can contribute to their learning through avoiding hunger and enhancing cognitive performance. In our previous study, we reported that the SFP promoted multiple academic outcomes among socio-economically disadvantaged children^(22)^. Similarly, studies conducted in the southern part of Ethiopia in Dara District^(23)^ and Borecha District^(24)^ also showed SF increases rates of enrollment and academic performance while reducing drop-outs and absenteeism.

Among the challenges of the SFP, first and foremost, the study showed that most of the schools with the feeding programs do not have access to regular and safe drinking water sources. As KIs explained, 75 % of schools have no water source inside the school and those schools which have potential water sources did not have regular water supply. As a result, students and paid labourers were obliged to fetch water by travelling for long distances. A similar study conducted in Jigjiga zone, Somali regional, Ethiopia, showed that problems related to water are the most serious ones^(19)^. A retrospective study conducted in Ghana likewise showed an estimated 43 % of schools being supported by an SFP did not have access to good, safe and regular supplies of water and therefore students wasted their valuable time by going long distances to fetch water^(25)^. In addition, another study conducted in northern Ghana indicated that water was a major problem in the schools, and therefore, the schools had to buy water from tanker drivers for cooking^(26)^.

Secondly, late delivery of the food to schools is a serious problem confronting the program. Even though the HG-SFP is expected to improve food delivery time to the school, late delivery of food to schools was reported by all school directors in the study area. Many factors contributed to the late delivery as explained by an education delegate. For example, the farmer's union/cooperative lacks experience in processing and dispatching huge amounts of food, and they do not have standard milling facilities. Bureaucratic issues in procurement procedures also contributed to late delivery. Consistent with this, other studies conducted in Ghana^(26)^, Jigjiga zone^(19)^ and Southern Ethiopia^(23)^ showed late delivery to be a challenge for SFPs.

Thirdly, problems of kitchens, storage, canteens, cups, plates and spoons are also main challenges for the SFP in the study area. Our interviews with school directors indicated that about 75 % of the schools in the study area are not equipped with suitable kitchen structures and facilities to cook food in a hygienic and safe environment. This may influence students’ health negatively. In addition, most of our study participants stated that many schools cook the food under small open structure shelters. Sometimes during the rainy season, the meal of the day is skipped due to the lack of a place to cook. Similar to our findings, a study from Ghana found that the schools did not have well-built kitchen structures and facilities to cook food^(25)^. Another study in Ghana also reported that many schools did not have kitchens to prepare food for students; instead, they cooked under trees and classroom verandas^(26)^. A similar study in Jigjiga zone, Somali region showed that schools’ kitchens are not properly equipped^(19)^.

Teachers and school directors explained that due to the lack of eating rooms/cafeterias in most schools, students eat their meal under trees or in classrooms. The latter may affect teaching because the feeding makes the classroom unclean as well as making their reading and writing materials dirty. This finding is similar with a study in the Kwaebibirim district of Ghana^(27)^.

As FGD discussants and KIs explained, most of the schools do not have enough plates, cups and spoons for students to use. Therefore, two or three students are forced to share food in a single plate or they must wait for a longer time. This affects class time negatively because of extended lunch break time. A study conducted in northern Ghana reported that 60 % of schools studied did not have these materials and some schools even did not have any plates, cups and spoons. Therefore, students had to come to school with their own set of knives, forks and plates^(26)^.

Another challenge explained by study participants is that most of the schools have poor storage facilities. Because of the lack of enough ventilation in the storeroom, the moisture content of the food increases which in turn favours microbial growth and spoilage. Due to this problem, the tendency for food items to be spoiled is very high. Furthermore, both KIs and FGD participants explained that the food delivered to schools was not properly sealed. Therefore, poor packaging and storage cause cross contamination with surrounding environments that leads to undesirable health hazards.

Additionally, cooks employed have not received formal training in sanitation and hygiene. According to reports from cooks, all employees and volunteer mothers in the cafeteria have not received any formal training about sanitation and hygiene. They only get instruction from focal persons and school directors. This may affect the quality of meals being served to the students, because cooking for large numbers of people is not the same as cooking for a household. A hygienic and safe environment is critically needed. Similarly, a study from Ghana reported that about 22 % of the cooks employed in the schools did not have health certificates and had received no kind of training in nutrition and hygiene^(25)^.

The cooks faced a high workload. Almost all school directors in the study area explained that the cooks faced a very heavy workload because the cereals delivered to schools for ‘Nifro’ took a long time to cook and required much firewood and water. In addition, mothers who worked as cooks explained that poor-quality grains delivered to the school affected their cooking by taking more time to clean the discoloured and damaged grain. This also affects the amount of food that can be prepared for the children because so much damaged and unclean grain has to be discarded.

Finally, the present study was conducted before the COVID-19 pandemic and the closure of the schools might have caused additional challenges to the beneficiary students^(28)^. A study conducted in Addis Ababa showed that the suspension of school meals during COVID-19 affected the progress made by the program and students. Before the school closure, children used to eat breakfast and lunch and then take the leftover for their snacks in the evening.

### Limitation of the study

The study did not capture the perspective of some of the national partners in the SFPs, such as the Ministry of Education, and the international partners, including FAO and WFP.

## Conclusion and recommendation

This qualitative study confirmed that HG-SFP has resulted in better quality education by improving enrollments, academic performance and attendance. In addition, students liked the home-grown meal menu and the program saved the parents’ money and time as a result of the food provided. With regard to SFP challenges, lack of permanent clean water provision, delay in ration delivery, poor-quality food provision, lack of necessary infrastructure for the program, and lack of training in sanitation and hygiene for cooks were among the major challenges discussed.

To improve the quality of the SFP, all schools need sufficient clean water provision for cooking and cleaning. Food storage facilities should be ventilated and protected from insects, rodents and animals. Kitchens and lunchrooms should be properly constructed. Basic cooking facilities including kitchen utensils, cooking pots and serving dishes should be provided to all schools in the required amount.

In order to increases the benefit of school food in terms of some key micronutrients, schools or nearby farmers should be encouraged to grow micronutrient-rich local foods such as nutritious leafy fresh vegetables and fruits which are important sources of vitamins and minerals.

To improve quality and hygiene challenges, all actors in the supply chain should have all the required basic knowledge to apply quality and safety standards. In addition, the quality of food should be checked for any signs of infestation and expiration during delivery and before consumption.

Finally, SFPs are not stand-alone interventions. They require inputs from other institutions in terms of both educational and health/nutritional needs. Therefore, strategic and operational barriers to the program need high-level attention in order to make the HG-SFP more successful in Sidama Region. In addition, the UN Sustainable Development Goal (SDG) 2 (‘Zero Hunger’) might help the country by eradicating hunger, achieve food security and improved nutrition and promote sustainable agriculture.

## Supplementary material

For supplementary material accompanying this paper visit https://doi.org/10.1017/jns.2022.77. click here to view supplementary material
