# Supplementary material for: Successes and challenges of the Home-grown School Feeding Program in Sidama Region, Southern Ethiopia: a qualitative study
Source: J Nutr Sci. 2022 Sep 30;11:e87. doi: 10.1017/jns.2022.77 (PMC9554426; doi:10.1017/jns.2022.77)
Supplement: Supplementary file 1 [file S2048679022000775sup001.docx]

| **Supplementary Tables**  **Table S1. Guide for focus group discussions and interviews** | |
| --- | --- |
| **Key Informant Interview Guide: School Feeding Program School Directors** | |
| 1 | Do you think that the economic activity in which parents are involved can affect students’ class attendance? Are parents in the area willing to send their children to school? |
| 2 | Can you tell me how the SFP was initiated? |
| 3 | Which organizations support the SFP? |
| 4 | How do you provide food to school children? |
| 5 | Who is responsible for cooking and monitoring the feeding sessions? |
| 6 | Does every child in the school participate in the SFP? |
| 7 | Do think that the SFP improves students’ class attendance and school enrollment? (Probe for details)And how? |
| 8 | What are the challenges of the SFP in your school? (Probe for details) |
| 9 | In general what should be done to improve the SFP as well as students’ class attendance and academic performance? Explain your ideas |
| **Key Informant Interview Guide: Woreda/district delegates** | |
| 1 | Can you tell me how the school feeding program (SFP) was initiated in the district? |
| 2 | Which organizations support the program? |
| 3 | Where do you get the food which you distribute to each school |
| 4 | How do you distribute food to each school? Do you have any mechanism to check the quality of the food that distributed to the schools? |
| 5 | Do you have any supervision programs? |
| 6 | Does every child in a SFP school participate in the feeding program? Do you think the food which you distribute is enough for the whole academic year to feed each child? |
| 7 | Do think that the SFP improves students’ class attendance and school enrollment? (Probe for how) |
| 8 | What are the main benefits of the SFP in your district? |
| 9 | What are the challenges for the SFP in your district? |
| 10 | In general what should be done to improve the SFP, and students’ class attendance and academic performance?  (Probe for details)Explain |
| **Key Informant Interview Guide: Teachers** | |
| 1 | Can you tell me how the school feeding program (SFP) was initiated in the school? |
| 2 | What factors affect children’s enrollment and dropout in your school? |
| 3 | What factors affect children’s enrollment and dropout in your school? |
| 4 | Do you think that livelihood activities of their parents affect student’s education? How? |
| 5 | Do you think SFP help students? How? |
| 6 | What are the benefits that the student got from SFP? |
| 7 | How do you contribute in assisting the school feeding program? |
| 8 | What factors affect the effectiveness of SFP implementation? / What are the main challenges of the program? |
| **Focus Group Discussion Guide for Parents of Students** | |
| 1 | What are your sources of livelihood? |
| 2 | What is your perception towards education and its importance?  Are you willing to send your children to school including girls? Do you think that education will benefit you and your children in the future? |
| 3 | Do you think that your sources of livelihood activities affect children’s education? What are the main activities that children’s perform in the household? |
| 4 | Do you know about the SFP in the school? Do you know why the school included in SFP? |
| 5 | What is your contribution in assisting the school feeding program? |
| 6 | What is the benefit of SFP to children’s and parents? |
| 7 | What problems affect SFP implementation in the school? |
| **Focus Group Discussion Guide for Students** | |
| 1 | What is your perception towards SFP and do you think SFP help you? How? |
| 2 | Are you happy with program? Do you like the school food that provided for you? |
| 3 | What are the benefits you have got from SFP? |
| 4 | How do you contribute in assisting the program? |
| 5 | What are factors challenges your feeding program? |
|  |  |

**Table S2.1. Themes and illustrative quotes about school feeding program and its success**

| **Themes** | **Data source** | **Times mentioned** | **Illustrative quotes** |
| --- | --- | --- | --- |
| How the SFP was initiated | KII | 20 | *“…School feeding program was initiated in our school in 2005. Because of our district is affected by drought.”*  ‘’…. *Due to the droughts,* *there was pronounced disappearance of students from school. That is why school feeding was started in our school.”* |
|  | FGD | 45 |  |
| Organizations that support the SFP | KII | 21 | *“… World Food Program supports the feeding program in our school’’* |
| How food is provided | KII | 21 | *“…the World Food Program trained us to provide a meal prepared from 100g dry whole grain maize, 50g beans, 10g vegetable oil and 5g iodized salt per each student.*  *“... In our school we have committee established by school to provide the food daily to students. And the focal person checks whether or not the exact amount of food is prepared based on the program and checks how the cook cooks the food and delivers to the students. Hmm……. In addition teachers also participate in facilitating the feeding session.* |
|  | FGD |  | *“… After the cereal is taken from the store based on the number of students, we cook the food and deliver to the students. And most of the time the students eat the food at class break times.”* |
| Responsible for cooking and monitoring | KII | 21 | *“…in the begging of the program, volunteer mothers from the community cooked the food. Currently we have employed cooks in our school.”*  *“The cooking session is monitored by the committee members organized by the school. The assigned committee member for that day monitors the overall activities of the program.”*  *“In our school we have volunteer mothers from the community that participate in the feeding session. In addition we have two paid cooks participate in the cooking. The coordinator and the school principal monitor the feeding session.’’* |
| Every child eats the school meal | KII | 21 | “*…yes, the school food is prepared and delivered to the students in all school days, unless otherwise we face any problem, hum… most time we manage it by buying water from nearby kebeles, from river or springs”.* |
| Alleviate short-term hunger, | KII | 1 | *“…… in our school there are some children who come from remote and distant areas by walking 2-3 hours every day. When they arrive to school they get very hungry and this program helps to get their lunch.”* |
| Increase enrolment, attendance and decrease gender disparity | KII | 21 | *“….. Before the SFP was launched in our school, we had observed that many children would not come to our school because the children would participate in different household activities to help their parents, especially female students. Hum mm ….. Currently this situation has considerably been improved and the feeding program is very helpful to have students in the school”*  *“… I think the feeding program is an important strategy to attract students to school. Compared to the previous years, the number of students has increased and the number of students who miss class has decreased. And even students from other kebeles frequently requested us to join this school because of the meals we provide.”*  *“……….since the SFP has been launched in our school, we have observed an increase in the number of children attending in our school. The parents are very happy to send their children to the school due to the food. “* |
|  | FGD | 1 | *“….. My son is learning in this primary school, and most of the time I don’t have anything to give him at home as breakfast. Before the SFP started, my son frequently had missed the class preferring to do work for money/food than attending school.”* |
| Happy with the food | KII | 16 | *“…. School children liked the existing HG-SFP menu. The previous menu (porridge made from soya blend) was less liked by the students.”*  *“….. Students showed their lack of interest to the previous menu by refusing to be served or by leaving significant amount of left-over food on their plates.”* |

**Table S2.2. Themes and Illustrative quote in the challenges of school feeding program**

| Themes | Data source | Times mentioned | Illustrative quote |
| --- | --- | --- | --- |
| Water problem | KII | 16 | *“…We don’t have water in our school compound, even in the near distance to the school. For these reasons we required each student to bring a bottle of water from his/her home.”*  *“….We don’t have budget to buy water daily to cook food for thousands of students. Hum... in addition the food needs much water and firewood to be cooked, especially the “Nifro” because it is made from dry maize and beans.”*  *“…Sometimes when there is no cooking water, the school feeding program is skipped.”*  *“…we do have water supply facilities in our school. But there is mostly no water in the lines. And an often time there is no water supply for about one month. When we buy water, filling a 20 liter water-containing jar costs us about 5 birr ( 0.16 USD).”* |
| Cooking wood problem | KII | 16 | *“..Previously the students brought us wood. But by now they are not doing so. Therefore, we are buying wood.”*  *“The food is cooked over four hours. Since the cereals are dry it consumes a lot of water and fire wood to make the food ready. To buy a lot of fire wood we don’t have budget.”*  *“…… we get fire food from the community. Students’ parents and the kebele leader are responsible to bring the fire wood to our school but it is not enough and sometimes we are forced to buy fire wood.”* |
| *food delivery delay* | KII | 20 | *“… yes, we have a problem of food delivery delay. For this year the cereals were provided to us in November. We received the food through the farmer’s union”* |
| *Problem of cereal quality* | KII | 20 | *“… We had a problem of cereal quality at the time of delivery. We received the food through the farmers union and they delivered some already spoiled cereal packages.”*  *“… On average more than 10 kg of waste is separated from a 50 kg cereals bag... and also the bag weight is not always constant as 50 kg, not properly sealed and expiry date is not labeled.”*  *“… Poor quality of the cereal delivered to our school has forced us to use more numbers of cooks in our school to separate the unwanted spoiled grain from the cereal.”* |
| Lack of kitchen utensils (cooking pots and serving dishes) | KII | 20 | *“….. Because we do not have enough dishes compared to the numbers of children. For example there are 680 students in a single shift of half day. But the numbers of dishes we do have are around 250.”*  *“…. we deliver food to students by coupling 2 or 3 students together to share from one plate.”*  *“…… schools did not have adequate stock of plates, cups, and spoons. As a result pupils eat in turns, and these prolonged lunch breaks reduce the time children spend in class”.* |
| The amount of food is not enough | KII | 20 | *“…the World Food Program trained us to provide a meal prepared from 100g dry whole grain maize, 50g beans, 10g vegetable oil and 5g iodized salt per each student. … But we don’t provide them this amount actually because the amount of the food which we received is not enough to do this daily”*  *“… The amount of the cereals allocated for the academic year is not enough. For these reasons, we are unable to feed the children by the recommended amount.* |
| Problem in foods storage facilities | KII | 8 | *“Our store is not ventilated. As a result maize and beans were infested with pests.”* |
| lack of training in sanitation and hygiene | KII | 16 | *“…… our cooks have not received any formal training about hygiene and sanitation since they started the job, instead they are relying on the instructions of their supervisors and their own household kitchen experiences over the years.”* |
| Permanent kitchen structures | KII | 8 | *….. Our schools did not have permanent kitchen structure. We cook under temporary structures. When it rains, we face great challenges to cook the food.* |
